# The Impact of Serum/Plasma Proteomics on SARS-CoV-2 Diagnosis and Prognosis

**DOI:** 10.3390/ijms25168633

**Published:** 2024-08-08

**Authors:** Maura D’Amato, Maria Antonietta Grignano, Paolo Iadarola, Teresa Rampino, Marilena Gregorini, Simona Viglio

**Affiliations:** 1 Department of Molecular Medicine, University of Pavia, 27100 Pavia, Italy; maura.damato90@gmail.com (M.D.); simona.viglio@unipv.it (S.V.); 2 Unit of Nephrology, Dialysis and Transplantation, IRCCS Policlinico San Matteo Foundation, 27100 Pavia, Italy; mariaantoniett.grignano01@universitadipavia.it (M.A.G.); t.rampino@smatteo.pv.it (T.R.); marilena.gregorini@unipv.it (M.G.); 3 Department of Biology and Biotechnologies “L. Spallanzani”, University of Pavia, 27100 Pavia, Italy; 4 Department of Internal Medicine and Therapeutics, University of Pavia, 27100 Pavia, Italy; 5 Lung Transplantation Unit, IRCCS Policlinico San Matteo Foundation, 27100 Pavia, Italy

**Keywords:** COVID-19, serum, plasma, proteomics, multi-omics, LC-MS

## Abstract

While COVID-19’s urgency has diminished since its emergence in late 2019, it remains a significant public health challenge. Recent research reveals that the molecular intricacies of this virus are far more complex than initially understood, with numerous post-translational modifications leading to diverse proteoforms and viral particle heterogeneity. Mass spectrometry-based proteomics of patient serum/plasma emerges as a promising complementary approach to traditional diagnostic methods, offering insights into SARS-CoV-2 protein dynamics and enhancing understanding of the disease and its long-term consequences. This article highlights key findings from three years of pandemic-era proteomics research. It delves into biomarker discovery, diagnostic advancements, and drug development efforts aimed at monitoring COVID-19 onset and progression and exploring treatment options. Additionally, it examines global protein abundance and post-translational modification profiling to elucidate signaling pathway alterations and protein-protein interactions during infection. Finally, it explores the potential of emerging multi-omics analytic strategies in combatting SARS-CoV-2.

## 1. Introduction

SARS-CoV-2, a highly infectious virus belonging to the coronavirus family and associated with severe acute respiratory syndrome (SARS) causing COVID-19, was first identified in Wuhan, China, in late 2019 and rapidly spread worldwide [1]. Given its high transmission rates and the continuous emergence of novel variants carrying mutations in the spike protein, rapid detection of viral infection has become paramount in damming the pandemic [2,3,4,5,6]. Early detection not only aids in the isolation of asymptomatic carriers, who unwittingly greatly contribute to transmission, but also facilitates prompt interventions to reduce severe complications [7,8,9,10]. Currently, due to its advantages, which include minimal sample requirement, rapid response time, and high accuracy compared to alternative methods, reverse transcription-polymerase chain reaction (RT-PCR) stands as the predominant diagnostic tool for COVID-19 [11]. However, RT-PCR has limitations such as sensitivity to conditions, potential for false negative/positive results, and limitations in detecting early and late-stage infections [12]. Moreover, its equipment can be costly, and laboratory operators require specialized training on how to use the instrument [13]. To complement PCR, rapid and precise diagnostic methods would be helpful to prevent transmission and enable early intervention that can reduce the risk of developing severe complications. Nanotechnology-based diagnostics and proteomics have emerged as promising avenues [14]. Proteomics, in particular, is able to characterize molecular alterations, thus offering insight into pathogenic mechanisms. The identification of potential diagnostic markers may produce qualitative/quantitative information useful to diagnose a disorder and/or to monitor the activity of therapeutics. In this perspective, this approach holds promise to be an excellent tool in the service of precision medicine [15,16]. Liquid chromatography coupled with mass spectrometry (LC-MS) enables the precise detection and quantification of hundreds to thousands of proteins, providing a comprehensive view of COVID-19’s impact on molecular pathways. Since the pandemic outbreak, the effects of COVID-19 infection on the protein profile of human fluids have been widely examined, and several proteomic approaches for rapid detection, determination of disease severity, and prognosis of SARS-CoV-2 have been developed [17,18]. These data will improve the strategies that can be adopted in disease control and treatment management of patients. However, despite significant research efforts, the correlation between protein changes and COVID-19 severity remains elusive due to sometimes conflicting findings across studies.

This article aims to examine proteomic investigations of blood plasma/serum during the years 2020–2023, assessing whether the substantial global research effort on the COVID-19 virus has fulfilled its promises. Through critical analysis, this report seeks to elucidate the role of proteomics in the identification of prognostic markers for efficient COVID-19 detection and in predicting disease outcomes.

Understanding the current state and challenges of serum/plasma analysis will inform future research directions in this emerging field.

## 2. The Use of Serum/Plasma for Investigating COVID-19

During the peak of the pandemic, nasopharyngeal epithelial swabs emerged as pivotal tools for the detection of the presence of the pathogenic virus in the mucosa and saliva of COVID-19 patients. Despite their utility, only a few preliminary proteomics studies have focused on the identification of biomarkers and potential drug targets of COVID-19 using this specimen [19,20,21]. The primary target for biomarker discovery remains blood serum or plasma, owing to the rich diversity of proteins and biological molecules they contain, which make them more attractive than any other source for proteomic investigations. Nevertheless, serum/plasma analysis comes with its own set of challenges. First of all, standardizing sample preparation and handling poses difficulties, and detection of proteins spanning a wide dynamic range (9–10 orders of magnitude) can have severe limitations. Furthermore, the divergence in protein composition between plasma and serum raises questions about which fluid is more informative for proteomic analysis.

**Figure 1:**
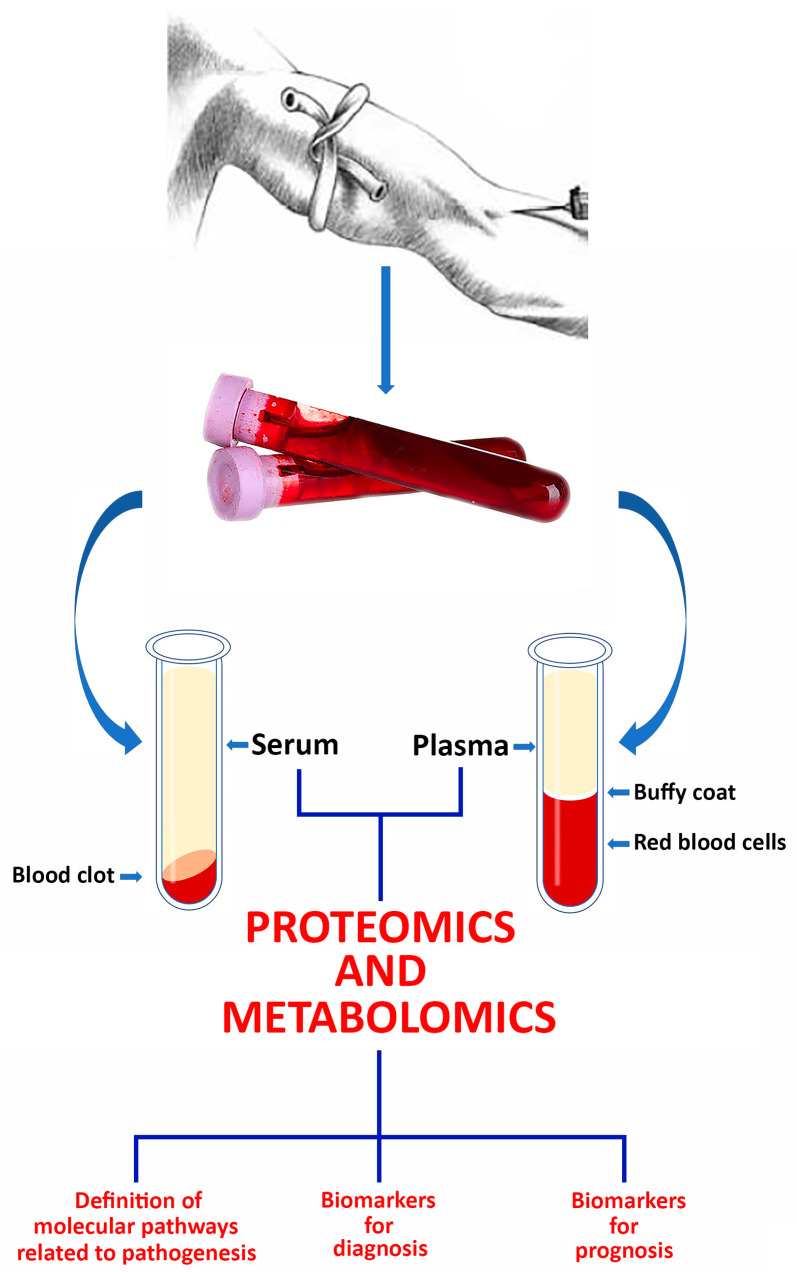
Cartoon schematizing the applications of proteomics in COVID-19 research considered in this report.

Despite these challenges, blood proteomics holds immense promise for both identifying diagnostic and prognostic markers and shedding light on disease mechanisms. Numerous studies have highlighted that alterations in human plasma/serum protein levels reflect pathophysiological changes caused by various diseases, including viral infections, making them viable targets for disease diagnosis and prognosis [22,23,24]. The applications of proteomics in COVID-19 research considered in this report are schematized in Figure 1.

## 3. Methodology Followed for the Preparation of the Current Article

Since COVID-19 emerged towards the end of 2019, our focus for this article spans from the beginning of 2020 to the present day. We conducted thorough research using databases such as PubMed, Web of Science, and Scopus. To ensure a comprehensive review of the literature, our search terms included “serum proteomics”, “plasma proteomics”, and “LC-MS/MS” along with the acronyms SARS-CoV-2/COVID-19. We exclusively considered peer-reviewed research articles and systematic reviews published in international journals and screened them for relevance. Due to the urgent nature of the pandemic, we found a significant number of contributions. We meticulously analyzed the full texts of selected papers and removed duplicates or redundant content. In total, 120 original studies detailing the application of serum/plasma proteomics in COVID-19 patient cohorts met our eligibility criteria. Among these, our emphasis was on those specifically focused on the identification of biomarkers useful in the prognosis/diagnosis of the disease. A list of additional references that could be complementary to the content of the current review article is also reported in the References section [25,26,27,28,29,30,31,32,33,34].

## 4. Serum/Plasma Proteomics for the Identification of Biomarkers/Predictors of the Disease

The current gold standard for diagnosing COVID-19 involves detecting viral RNA in respiratory samples using the reverse transcriptase-polymerase chain reaction (RT-PCR) test. While proteomics and metabolomics offer valuable insights into the molecular mechanisms of COVID-19, further research and validation are needed to determine the clinical utility of their results in diagnosis and prognosis of this disease. Nevertheless, efforts are underway to identify serum prognostic biomarkers that can promptly identify patients in need of immediate medical attention and estimate associated mortality rates. Numerous research teams are employing proteomics to identify markers of the disease or indicators of its severity and progression, with much of this research focusing on serum/plasma samples.

An experimental approach based on enzyme-linked immunosorbent assay (ELISA) and capillary gel electrophoresis coupled with laser-induced fluorescence detection (CGE-LIF) was used by Beimdiek et al. [35] to identify key biomarkers indicative of COVID-19 severity. Notably, patients exhibited significantly lower concentrations of disintegrin-like metalloprotease with thrombospondin type-1 motif 13 (ADAMTS13) and albumin compared to controls. Conversely, several inflammatory proteins were increased in patients relative to healthy individuals.

Lee et al. [36], using a liquid chromatography-electrospray ionization-quadrupole-Orbitrap mass spectrometry (LC-ESI-Q-Orbitrap-MS) approach, identified immunoglobulin lambda variable 3-19 (IGLV3-19) and basonuclin zinc finger protein 2 (BNC2), two proteins involved in humoral immune response, acute phase reaction, lipid metabolism, and platelet degranulation, as novel potential prognostic markers for severe COVID-19. Additionally, they observed longitudinal changes in other proteins as COVID-19 progressed towards severe stages, thus providing promising insights into the host response to SARS-CoV-2 infection. In a similar longitudinal study, Geyer et al. [37] used LC-ESI-Q-time-of-flight (TOF)-MS to compare proteomic profiles of serum samples from COVID-19 patients with PCR-negative symptomatic controls. They noted significant protein alterations between the two groups, with a decline in proteins associated with the innate immune system early in the disease course and a progressive increase in regulatory proteins involved in coagulation.

A different approach based on chemiluminescence immunoassay (CLIA) and nano-LC coupled with a hybrid Q-Orbitrap was applied by Liang et al. [38] to analyze serum IgM and IgG in COVID-19 patients, categorized into four groups based on serological patterns. Their findings suggested that high IgM titers might not be advantageous for COVID-19 recovery. Chen et al. [39], using ESI-Q-TOF-MS, profiled the proteome of serum samples from COVID-19 patients at disease onset and recovery stages. The authors highlighted prolonged disruptions in cholesterol metabolism and myocardial function, particularly in critically affected patients.

Li et al. [40] analyzed sera from patients with long-term persistent (LTP) infection, including those without hypertension (LTP-NH), using an ultra-high-performance (UPLC)-ESI-Q-MS. GO and KEGG analyses revealed a significant enrichment in “coagulation” and “immune response” proteins in both LTP and LTP-NH groups, thus highlighting the potential therapeutic significance of these molecules for managing LTP patients. Yan et al. [17] addressed the challenge of false negatives in COVID-19 diagnosis by proposing high-throughput serum peptidome profiling as an efficient alternative. Their analysis of sera from patients and controls enabled the development of a model with promising capabilities for large-scale screening and diagnosis of COVID-19.

n-LC-ESI-TripleTOF-MS was used by Zhang et al. [41] to analyze serum proteomes from severe and non-severe COVID-19 patients, non-COVID subjects with flu-like symptoms, and healthy controls. Among the numerous dysregulated proteins detected across five stages of disease progression, four were highlighted for their crucial roles in host defense and their influence on disease severity and prognosis. Integrating these insights with key clinical metrics, they developed two machine learning models to complement swab RT-PCR tests in monitoring COVID-19 progression. Similarly, Vollmy et al. [42] used an HPLC system coupled with an Orbitrap MS in data-independent mode (DIA) to investigate the serum proteome for markers predictive of mortality among patients with moderate to severe COVID-19. They focused on serum proteins exhibiting differential abundance between survivors and non-survivors. By defining an optimal panel of nine proteins for mortality risk assessment, the authors demonstrated its reliability in predicting mortality across independent cohorts and different laboratory settings.

Vaz-Rodrigues et al. [43] used nLC-ESI-TripleTOF-MS to analyze serum from COVID-19 patients both before and after vaccination, aiming to develop personalized nutrition protocols using prebiotics or probiotics for COVID-19 management. The fluctuations in acute-phase protein levels observed appeared to be correlated with disease severity and patient nutritional status, suggesting their potential utility in predicting prognosis for both vaccinated and unvaccinated individuals with COVID-19.

Villar et al. [44] also applied nLC-ESI-TripleTOF-MS to examine serum protein profiles across different cohorts. Out of 189 identified proteins in serum samples, four were highlighted as potential prognostic biomarkers. Kimura et al. [45] introduced an innovative proteomic approach to identify serum proteins linked to COVID-19 prognosis. They analyzed immunodepleted serum samples from severe COVID-19 patients using LC-ESI-Q-Orbitrap-MS in DIA mode, identifying 27 proteins differentially expressed between subjects with adverse or favorable prognoses. Di et al. [46] applied a LC-Orbitrap-MS platform for a quantitative proteomic analysis of sera from severe and moderate COVID-19 patients, aiming to identify biomarkers predictive of disease progression. Notably, D-dopachrome decarboxylase (DDT) played a pivotal role in the inflammatory cascade promoting cytokine storm syndrome, and increased myoglobin levels indicated kidney injury or dysfunction [47,48].

A hydrophilic interaction liquid chromatography (HILIC)-ESI-Q-Orbitrap-MS approach to uncover molecular patterns predictive of clinical outcomes was applied by Buyukozkan et al. [49]. The analysis of serum samples from COVID-19 patients revealed significant alterations in proteins involved in various metabolic pathways consistent with previous studies [50,51,52]. To distinguish high-risk (hospitalized) and low-risk (outpatient) COVID-19 cases, Lazari et al. [53] used a platform combining machine learning (ML) and MALDI-TOF-MS to analyze C18-fractionated plasma samples, identifying intact and truncated forms of serum amyloid proteins A-1 (SAA1) and A-2 (SAA2), known biomarkers for viral infections in the acute phase.

Fu et al. [54] introduced a novel analytical methodology integrating the high sensitivity of immunoprecipitation (IP) with nLC-ESI-Q-Orbitrap-MS. This approach enables the sensitive and selective quantification of SARS-CoV-2 protein antigens and anti-SARS-CoV-2 immunoglobulins in blood serum or plasma. Gutmann et al. [55] evaluated circulating SARS-CoV-2 RNA (RNAemia), establishing a correlation between RNAemia in COVID-19 ICU patients and increased mortality risk. Additionally, serum and/or plasma proteomic analysis, using nLC-ESI-Q-Orbitrap MS, identified mannose-binding lectin 2 (MBL2) and pentraxin-3 (PTX3) as positively associated with mortality, suggesting their involvement in the innate immune system’s complementary pathway. To rapidly diagnose COVID-19 in plasma samples, Delafiori et al. [56] developed a methodological approach based on ESI-Q-orbitrap-MS. They identified seven pivotal molecules belonging to the glycerophospholipid class, along with additional molecules categorized as sterol lipids, fatty acids, sphingosine, and a purine metabolite, all playing crucial roles in the disease’s mechanisms.

Kimhofer et al. [57] employed a multiplatform metabolic phenotyping strategy based on NMR spectroscopy and UPLC-ESI-TripleQ-MS to investigate the metabolic impact of SARS-CoV-2 infection on human plasma. Using orthogonal projections to latent structures (OPLS) analysis, they constructed a hybrid nuclear magnetic resonance (NMR)-MS model that facilitated detailed metabolic differentiation between the groups and elucidated their biochemical relationships.

The use of UPLC coupled with a TripleTOF MS enabled Messner et al. [58] to identify 27 potential biomarkers, whose expression varied with disease severity, in serum and plasma from COVID-19 patients. Their proteomic analysis highlighted the involvement of complement factors and the coagulation system in the host response to COVID-19. Di Flora et al. [59] employed UPLC coupled to an ESI-Q-TOF mass spectrometer to identify proteins associated with inflammation, immune response/complement system, and blood coagulation in plasma samples from 163 COVID-19 patients, suggesting that these proteins offer potential prognostic value upon hospital admission.

The analysis of plasma proteomic profiles from hospitalized COVID-19 patients, along with clinical data from a large patient database, enabled Meizlish et al. [60] to suggest a pivotal role for neutrophil activation in severe COVID-19 pathogenesis. In fact, detection of neutrophil activation as early as the first day of hospitalization preceded critical illness onset, indicating its potential for predicting clinical decompensation and mortality risk. Intriguing research by Beltrami et al. [61] combined Proximity Extension Assay (PEA) technology [62] with ML to identify the strongest predictors of disease severity by aggregating numerous parameters with plasma proteomics data.

PEA was also employed by Iosef et al. [63] to analyze plasma proteome from long-COVID patients in a study aimed at elucidating underlying mechanisms and informing prognostic and therapeutic strategies. The data obtained showed that long-COVID outpatients were characterized by neutrophil activation leading to extracellular trap formation. Furthermore, several markers identified suggest a vasculo-proliferative phenomenon in long COVID that may contribute to alterations in the organ-specific proteome. The same authors [64] used PEA to identify protein signatures associated with COVID-19 severity, mortality, and tissue recovery patterns, revealing a distinct plasma profile associated with COVID-19 that drives disease severity and unveils plasma biomarkers crucial for tailored therapeutics.

A correlation between mortality risk and the imbalance between type2/type 1 helper (Th2/Th1) cells has been established by Pavel et al. [65] by means of PEA. The analysis of sera from patients infected with SARS-CoV-2 suggested that all deceased patients evidenced a higher Th17/Th1 cytokine imbalance compared to survivors. Additionally, Th2/Th1 imbalance was higher in the sera of asthma patients at admission who did not survive COVID-19. Li et al. [66] evaluated the relationship between SARS-CoV-2 viremia, disease outcome, and proteomic profiles in a cohort of 300 COVID-19 participants from an emergency department. Using PEA for high-multiplex protein analysis and a quantitative reverse transcription PCR-based platform to measure SARS-CoV-2 viral load, they found that the cascade of vascular and tissue damage associated with SARS-CoV-2 plasma viremia could be a tool to predict COVID-19 disease outcomes.

A PEA-based methodological approach was also applied by Al-Nesf et al. [67] to analyze plasma samples from healthy controls and COVID-19 patients with varying disease severity. The proteins identified, implicated in metabolic processes primarily associated with immune, inflammatory, and infection pathways, were proposed by the authors as potential targets for candidate drugs aimed at preventing or treating severe COVID-19 complications. A UPLC approach coupled with ESI-Q-Orbitrap-MS was applied by Ciccosanti et al. [68] to perform an extensive proteomic analysis of plasma samples from a series of COVID-19 patients. The results highlighted notable increases in several proteins associated with the complement cascades in COVID-19 patients requiring ICU admission. Additionally, proteins involved in acute inflammatory responses, platelet function, and neutrophil activity showed progressive elevation in ICU COVID-19 patients compared to non-ICU cases.

Paes-Leme et al. [69] analyzed serum-derived extracellular vesicles (EVs) from COVID-19 patients by nLC coupled with ESI-Q-Orbitrap-MS to identify and quantify protein signatures that could provide insights into differences of disease severity. The main biological signatures in moderate/severe COVID-19 were associated with platelet degranulation, exocytosis, complement activation, immune effector activation, and humoral immune response. The message emerging from these data is that EV proteins may be relevant biomarkers of disease state and prognosis.

An interesting review article by Shengman et al. [70] describes the current understanding of COVID-19 in terms of the quantitative and qualitative proteomics of viral particles and host entry factors from the perspective of protein pathological changes in the organism following host infection.

A schematic summary of all articles commented above is shown in Table 1.

## 5. Use of Multi-Omics in COVID-19 Research

The data provided by proteomics (and other single omics technologies) typically allow a list of differences between healthy and disease groups to be assembled. While being interesting, this information may still be limited since it reflects reactive processes rather than causative ones [71]. Recent technological advances that have allowed different omics technologies to integrate each other have resulted in the development of a “multi-omics” approach that makes it possible to explore the entire pools of biological molecules present in an organism. Compared to single omics analysis, the advantage of integrating different omics data is not only to give insight into which biological pathways are different between the disease and control groups but also to obtain information about the original cause of disease. These data certainly provide researchers with a greater understanding of the mechanisms involved in complex human diseases. In particular, it is crucial to recognize for long-COVID patients that this syndrome comprises various clinical manifestations and necessitates a comprehensive, multidisciplinary approach tailored to the specific symptoms and their severity. Addressing this complexity represents the toughest challenge.

Among the reports found in the literature, the proteomics-metabolomics combination seems to be the most promising approach in studying COVID-19. The pioneers in this field were Shen et al. [72], who combined proteomics and metabolomics to unambiguously identify in the sera of severe COVID-19 patients protein and metabolite changes that might be used as potential blood biomarkers to evaluate the severity of the disorder. Spick et al. [73] applied quantitative metabolomics and DIA proteomics to investigate the interactions between COVID-19 and glucocorticoids used for treating patients requiring oxygen supplementation. Their findings, while providing further understanding of glucocorticoid action, also highlight that despite the broad omics dysregulation caused by COVID-19 infections, glucocorticoids do not play a pivotal role. These two techniques were also combined by Yan et al. [74] to compare quantitative serum proteomic and metabolomic differences in a subset of COVID-19 patients classified into high- and low-risk groups based on serum lactate dehydrogenase (LDH) concentration, a prognostic indicator of the disorder. Their results showed that serum LDH levels were associated with COVID-19 severity.

Yang et al. [75] applied quantitative proteomics and untargeted metabolomics to study COVID-19 complications in patients with pulmonary fibrosis. While imaging revealed lesions in the bilateral lower lobes and involvement in five lobes, molecular analysis indicated the involvement of important pathways in fibrosis progression. Li et al. [76] combined proteomics and metabolomics to analyze plasma samples from controls and COVID-19 survivors six months after hospital discharge. Gene ontology and enrichment analysis of the differentially expressed proteins and metabolites suggested that COVID-19 survivors still show persistent abnormalities in protein and metabolites plasma levels. Bi et al. [77] focused on proteome and metabolome analysis of urine and serum from patients and healthy controls. Their results demonstrated that the urinary proteome might add value in evaluating the clinical course of COVID-19.

To understand the effect of tobacco smoking on the development of COVID-19, Cui et al. [78] performed a comprehensive bioinformatics analysis on three serum proteomics and metabolomics databases. They investigated COVID-19 status, smoking status, and basic information of a population to analyze the interactions of proteins or metabolites. Based on the outcome of this survey, they observed that smoking contributes to an increased risk of progression from non-severe to severe COVID-19 by inducing the dysfunctional immune response. The importance of integrating proteomics and metabolomics data has been emphasized by Costanzo et al. [79] in a recent review article in which the authors point out the improvement of knowledge that can be acquired by combining these two techniques in finding new therapeutic solutions in the fight against COVID-19.

While the association of proteomics and metabolomics is the most commonly used multi-omics strategy, other approaches have also been explored in COVID-19 research. Kawasaki et al. [80] integrated the next-generation proteomics of serum extracellular vesicles with single-cell RNA sequencing (scRNA seq) of peripheral blood mononuclear cells. The aim of this study was to discover potential serum biomarkers and examine proteins associated with the pathogenesis of refractory COVID-19. The authors found that the expression of a single protein, MACROH2A1, was significantly elevated in refractory cases compared to non-refractory ones, thus suggesting its presence in extracellular vesicles as a potential biomarker of refractory COVID-19.

Sanchez et al. [81] employed a multi-omics approach with plasma from patients at various stages of COVID-19 severity to analyze the metabolic changes occurring during SARS-CoV-2 infection. Remarkably, the biomolecules related to COVID-19 severity were linked to specific metabolic pathways including mitochondrial dysfunction, lipid metabolism, amino acid biosynthesis, and coagulation. Understanding these disrupted molecular pathways, persisting beyond the acute phase, is crucial not only for discerning differences in recovery among SARS-CoV-2-infected individuals during COVID-19 progression but also for anticipating and comprehending the emergence of future pathologies, such as long COVID.

The aim of the multi-omics study carried out by Altendahl et al. [82] was to investigate the pathophysiology behind various clinical trajectories in pregnant patients diagnosed with COVID-19. Their results highlighted severe COVID-19’s association with distinct profiles characterized by significant proteomic and lipidomic signatures enriched for annotations related to complement and antibody activity. Conversely, the levels of serum protein, metabolite, or lipid metabolite in post-infection mild/moderate COVID-19 were not significantly altered compared to controls. Kugler et al. [83] undertook a multi-omics study to identify predictors of short-term COVID-19 disease progression during the acute phase in intensive care patients to support clinical decision-making. The plasma proteome, metabolome, and lipidome were determined for each sample by LC-MS analysis. Their study identified a predictor capable of forecasting worsening patient conditions up to five days in advance with reasonable accuracy. Interestingly, the predictor’s performance complemented clinicians’ capabilities to predict patient deterioration.

A schematic summary of all articles commented on above is shown in Table 2.

## 6. Did Proteomics Meet Expectations in COVID-19 Research ?

In the realm of medicine, there has historically been a remarkable delay between the findings of fundamental research and their translation into practical tools for clinical use. This gap arises from the inherent split between the objectives of basic research, whose purpose is to generate knowledge, and clinical practice, which aims to enhance patient well-being. Notably, during the early phases of the COVID-19 pandemic, the primary focus was on limiting the spread of the disease and minimizing the number of deaths. Consequently, only at a later stage was research focused on the identification of biomarkers for early diagnosis and treatment. Consequently, only a limited number of proteomics-based COVID-19 biomarker candidates have been translated to clinical application thus far. Linking the bench to the bedside as early as possible would allow the rapid transfer of new findings into clinical tools. The urgency of the pandemic’s initial phase, coupled with the complex clinical spectrum of COVID-19 and its multifaceted difficulties (including interindividual variability across demographics and comorbidities), further complicates the establishment of diagnostic criteria and treatment protocols. This complexity challenges the integration of research findings in clinical practice, hampering the timely translation of discoveries. Additionally, while little is still known about the long-term health effects of the virus (due to the short time since its emergence), the occurrence of new SARS-CoV-2 variants underscores the necessity of quickly assessing their potential severity. Despite these challenges, the global scientific community has maintained a high level of focus on COVID-19, resulting in a significant body of research in a relatively short timeframe, particularly in the field of proteomics. Early applications of proteomics, particularly in plasma/serum analysis, have primarily aimed at identifying novel markers to enhance disease understanding, diagnosis, prognosis, and monitoring of disease severity/progression. Notably, Messner et al. [58] pioneered a high-throughput platform for COVID-19 biomarker identification and disease progression analysis, which is not strictly usable in a research environment only but is a feasible approach that can be readily implemented in routine laboratory settings. This method, based on LC-MS technology, has demonstrated its reliability through meticulous optimization of all procedural steps, from sample preparation to chromatography, data acquisition, and processing. This has facilitated the identification of key proteins (complement factors, inflammation modulators, pro-inflammatory signaling, or proteins belonging to the coagulation system) associated with COVID-19 severity and complemented conventional clinical assays. Recent insights into the cytokine storm and immunosuppression observed in severe COVID-19 cases [84,85] have practical implications for treatment. Prompt identification of these biomarker signatures enables targeted interventions, potentially reducing mortality risk by addressing hyperinflammation using existing therapies. However, for biomarkers to be useful in the clinical setting, they must exhibit sufficient sensitivity and undergo rigorous validation. Challenges such as the dynamic range of protein concentrations, which can prevent the detection of less abundant proteins on many platforms, must be addressed through technological advancements and collaborative efforts to ensure the successful translation of proteomic discoveries into clinical practice.

Proteomic studies on plasma or serum can be significantly affected by the COVID-19 vaccination status of patients. This is because the immune responses of vaccinated and unvaccinated individuals differ. Vaccinated individuals, having pre-existing immunity from the vaccine, exhibit a more rapid and effective immune response to the virus. In contrast, unvaccinated individuals experience a naïve immune response, resulting in different kinetics and quality of response to the virus. Consequently, proteomic profiles between these cohorts can vary greatly in terms of the levels and types of proteins expressed in response to infection. Vaccines induce specific immune pathways that alter cytokine and chemokine levels and promote the formation of memory B and T cells, leading to the expression of different protein sets compared to those produced by naïve immune cells [86,87].

These differences are crucial, as they shape the overall plasma or serum proteomic landscape during infection. Furthermore, since vaccination typically reduces the severity of COVID-19, vaccinated individuals often show proteomic profiles with lower levels of inflammatory markers, reflecting a milder form of the disease. In contrast, unvaccinated individuals generally exhibit profiles indicative of severe disease and heightened inflammation. By carefully considering and controlling for patients’ vaccination status, researchers can more accurately deduce the effects of COVID-19 on the plasma or serum proteome. This approach enhances the understanding of disease pathophysiology and leads to more precise conclusions, potentially informing therapeutic interventions [86,87]. The relevance of putative biomarkers identified in the early stages of the COVID-19 pandemic for more recent virus variants is a nuanced issue. While some early biomarkers associated with the host immune response (i.e., markers of inflammation, cytokine levels, and certain acute-phase proteins) may still hold relevance, others may need adjustment to reflect the characteristics of new variants. In fact, mutations impacting viral behavior, such as transmission, virulence, and immune evasion, can alter the host’s immune response, potentially affecting the levels and types of biomarkers. On the other hand, new variants may present with different clinical manifestations or severity, leading to the identification of new biomarkers that are more indicative of the disease’s current state. Additionally, as mentioned above, immunity from vaccines or past infections can modify the body’s responds to new variants, further impacting the expression of biomarkers.

## 7. What Is the Added Value Provided by Multi-Omics?

It has been previously highlighted that COVID-19 is a complex disorder with a multifaceted etiology. Relying solely on a single layer of data may not sufficiently elucidate the various factors contributing to the development or progression of the disease. While proteomics on its own can offer valuable insights into the metabolic dysregulation associated with the disorder, integrating different omics methodologies is expected to provide more comprehensive mechanistic information of the disease. However, two pivotal questions arise: First, what additional value (if any) can integrated methodologies offer? Second, is there an optimal combination of methodologies? Drawing an analogy with chromatography, just as 2D chromatography separates components in a complex mixture more efficiently than 1D, combining complementary omics can delve deeper into unanswered questions. It is well known that COVID-19 research faces a significant challenge due to the high variability in patients’ immunological responses triggered by the infection [88]. This variability stems from diverse factors, including individual patient characteristics and various risk factors [88]. Addressing this challenge necessitates the use of technologies capable of accurately characterizing each patient’s specific features, thus facilitating tailored treatments. This aligns with the principles of precision medicine, wherein proteomics and other omics techniques prove instrumental. While proteomics exhibits substantial potential in exploring discordant responses to disorders among patients (as shown in previous paragraphs), it has its limitations. Metabolomics emerges as a more sensitive approach to depicting phenotypic alterations [89]. By representing the physiologic status of a given cell/tissue/organism, it provides crucial insights into the biochemical pathways involved in disease mechanisms [89]. Comprehensive information on the contribution of this methodology in the study of the metabolic/lipid dysregulation produced by SARS-CoV-2 infection can be found in a series of articles recently published by Bruzzone et al. [90,91]. Notably, given the complementary nature of the data that the combination of proteomics and metabolomics produces, this stands out as the most promising integration. This integration has been widely adopted in multi-omics investigations, particularly in the study of SARS-CoV-2 infection, as shown by the several examples that underscore the importance of this integrated strategy in unravelling the pathogenesis of this disorder [64,65,66]. While other omics combinations exist in the literature [61], they appear more as experimental exercises rather than methodologies ready for clinical application. It is essential to acknowledge that the application of these platforms in studying SARS-CoV-2 infection is still in its infancy. Challenges such as differences in techniques among laboratories and interindividual variability complicate data interpretation. Nevertheless, ongoing efforts hold promise for constructing an overall protein-metabolite regulatory network [92]. This will enhance our understanding of disparate biological pathways between patient cohorts and identifying potential disease markers.

## 8. Conclusions

Despite significant reductions in case numbers and death rates since its peak, COVID-19 continues to claim lives daily, underscoring the ongoing need for vigilance. The disorder is characterized by multi-organ damage resulting from systemic immunological disruptions believed to be mediated by the bloodstream. To deepen our understanding of the host response to SARS-CoV-2 infection, extensive analyses of circulating soluble proteins in the blood have been conducted using global proteomics.

As we enter the fifth year of the pandemic, MS-based proteomic studies on COVID-19 have proliferated globally. These studies reveal substantial alterations in the soluble blood proteome associated with the disease. Identifying specific proteins whose levels change during disease progression could provide critical insights into the underlying biochemical pathways involved. Historically, circulating biomarkers have played a crucial role in clinical decision-making for infectious diseases, aiding in the assessment of disease severity and treatment effectiveness.

For COVID-19, there remains a critical need to further identify biomarkers related to host response, organ involvement, and prognosis. MS-proteomics holds promise in identifying/quantifying these proteins and observing their differential expression. However, determining whether proteins identified as key drivers of pathological mechanisms can indeed define diagnostic criteria or tailor potential therapies is perceived as premature.

Methodological disparities among research groups in quantifying COVID-19-associated changes pose a significant challenge, hindering direct study comparisons. Moreover, variations in sample sizes within studies using the same methodology may yield equivalent results but lead researchers in divergent clinical decision-making directions. Nevertheless, the increasing integration of diverse omics data layers is facilitating the identification of potential causative changes and treatment targets. Accurate interpretation of omics findings stands to enhance our comprehension of the long-term health consequences of COVID-19.

## Figures and Tables

**Table 1:** List of articles dealing with proteomics of serum/plasma in COVID-19 patients.

Reference	Source	Subjects Investigated *	Proteomic Technique	Main Findings
[35]	Plasma (n = 30)	HC = 10 P = 20 (10 moderate and 10 severe)	Nephelometry ELISA CGE-LIF	COVID-19 affects the levels and glycosylation patterns of certain plasma proteins.
[36]	Serum (n = 137 plus C)	HC = n.r. ** P = 25 (13 moderate and 12 severe)	LC-ESI-Q-Orbitrap-MS	COVID-19 biomarkers involved in humoral immune response, interferon signaling, acute phase response, lipid metabolism and platelet degranulation have been identified together with 11 predictors of progression to severe form.
[37]	Serum (n = 458)	C = 262 (Patients with COVID-19-like symptoms negative to RT-PCR) P = 31	LC-ESI-Q-TOF-MS	A set of 20 biomarkers has been identified, including proteins related to the immune system, blood clotting, and lipid homeostasis.
[38]	Serum (n = 144 plus C)	C = n.r. ** P = 144	RT-PCR CLIA nLC-ESI-Q-Orbitrap-MS	High titers of IgM might not be favorable to COVID-19 recovery.
[39]	Serum (n = 26)	HC = 10 P = 16 (10 moderate and 6 severe)	LC-ESI-Q-TOF-MS	Prolonged disruptions in cholesterol metabolism and myocardial function are related to COVID-19 infection, especially in severely affected patients
[40]	Serum (n = 27)	HC = 15 P = 12 (5 LTP and 7 LTP-NH)	UPLC-ESI-Q-MS	In both cohorts, there was an increase in coagulation and immune-response proteins.
[17]	Serum (n = 416)	C = 152 (patients with flu-like symptoms, healthy controls, Tb patients) P = 146	MALDI-TOF-MS	MALDI-TOF-based serum profiling is a rapid and accurate method for the detection of COVID-19, with great potentials for screening, routine surveillance, and diagnostic applications
[41]	Serum (n = 275)	HC = 21 C = 24 (patients with flu-like symptoms) P = 144 (108 moderate and 36 severe)	nLC-ESI-TripleTOF-MS	Two machine learning models predicting nucleic acid positivity have been developed.
[42]	Serum (n = 99 plus C)	C = n.r. ** P = 33	LC-ESI-Q-Orbitrap-MS	Definition of a protein panel for mortality risk assessment
[43]	Serum (n = 142)	Pre-vaccine cohorts: 22 Post-vaccine cohorts: 120	nLC-ESI-TripleTOF-MS ELISA	COVID-19 disease prognosis can be predicted with serum nutritional biomarkers.
[44]	Serum	HC = 25 P = 95 (16 asymptomatic, 26 post recovery, 28 moderate, and 25 severe	nLC-ESI-TripleTOF-MS	The identification of prognostic biomarker proteins in SARS-CoV-2-host interactions
[45]	Serum (n = 71 plus C)	**Development group:** C = n.r. ** P = 10 **Validation group:** HC = n.r. ** P = 61 (15 adverse prognosis and 46 favorable prognosis)	LC-ESI-Q-Orbitrap-MS	High mortality risk is predicted by serum levels of two proteins closely linked to the pathogenesis of COVID-19.
[46]	Serum (n = 83)	**Development group:** P = 23 (8 moderate and 15 severe) **Validation group:** HC = 10 P = 50 (21 moderate and 29 severe)	LC-ESI-Q-Orbitrap-MS	Identification of a factors panel for predicting deterioration of moderate COVID-19 patients before symptoms manifest
[49]	Serum (n = 679)	C = 97 (patients with COVID-19 symptoms negative to RT-PCR) P = 330	HILIC-ESI-Q-Orbitrap-MS	Identification of distinct protein-metabolite cross talk related to immune modulation, energy and nucleotide metabolism, vascular homeostasis, and collagen catabolism
[53]	Plasma (n = 117 plus C)	C = n.r. ** P = 117	MALDI-TOF-MS nLC-ESI-Q-Orbitrap-MS	Increased levels of SAA1 and SAA2 proteoforms could be a measure of the increased severity of the disease.
[54]	Plasma Serum (n = n.r. *)	C = n.r. ** P = n.r. *	Immunoprecipitation nLC-ESI-Q-Orbitrap-MS ELISA	Immunoprecipitation-targeted proteomic assays could facilitate standardization of the existing serological tests.
[55]	Plasma (n = 474) Serum (n = 474)	C = 55 P = 123	RT-qPCR nLC-ESI-Q-Orbitrap-MS ELISA	COVID-19 ICU patients have a distinct proteomic pattern associated to mortality.
[56]	Plasma (n = 1004)	HC = 350 C = 23 (patients with COVID-19 symptoms negative to RT-PCR) P = 442 (246 moderate, and 191 severe)	ESI-Q-Orbitrap-MS	The proposed model demonstrated high accuracy in the diagnosis and risk assessment of COVID-19.
[57]	Plasma (n = 45)	HC = 25 P = 17	NMR UPLC-ESI-TripleQ-MS	Several aromatic amino acids and other metabolites were significantly altered, suggesting liver dysfunction, dyslipidemia, diabetes, and coronary heart disease risk. These findings confirmed that COVID-19 is a systemic disease affecting multiple organs and systems.
[58]	Plasma (n = 63 plus C)	**Development group** HC = n.r. ** P = 31 **Validation group** HC = 15 P = 17	UPLC-ESI-TripleTOF-MS	27 potential biomarkers of COVID-19 severity have been identified (complement factors, coagulation system components, inflammation modulators, and pro-inflammatory factors).
[59]	Plasma (n = 163 plus C)	HC = n.r. ** P = 163 (76 moderate, 56 severe, and 31 critic)	UPLC-ESI-Q-TOF-MS	Inflammatory, immune, complement systems, and coagulation proteins could be targets for appropriate therapies.
[60]	Plasma (n = 112)	**Development group** Cross-sectional cohort C = 13 asymptomatic patients P = 49 (40 ICU) **Validation group** Longitudinal cohort	Protein arrays ELISA scRNAseq	Data suggest a central role for neutrophil activation in the pathogenesis of severe COVID-19.
[61]	Serum (n = 160)	C = n.r. ** P = 160 (80 moderate and 80 severe)	PEA	Identification of 9 severe COVID-19 biomarkers and of 3 biomarkers linked to central nervous system pathologies, whose expression is decreased in severe forms.
[63]	Plasma (n = 66)	H.C. = n.r. ** P = 66 (22 long COVID, 22 moderate and 22 severe)	PEA ELISA	A vascular proliferative state associated with hypoxia inducible factor 1 pathway suggested progression from acute COVID-19 to long COVID. This may contribute to alterations in the organ-specific proteome, reflecting neurological and cardiometabolic dysfunction.
[64]	Plasma (n = 30)	HC = 10 C = 10 (COVID-19-negative) P = 10	PEA ELISA	COVID-19 resulted in reduced antigen presentation and B/T-cell function, increased repurposed neutrophils and M1-type macrophages, relatively immature or disrupted endothelia, fibroblasts with a defined secretome, and reactive myeloid lines.
[65]	Serum (n = 288 plus C)	C = n.r. ** P = 288 (21 with asthma)	PEA	Th2/Th1 interplay may affect patient outcomes in SARS-CoV2 infection. Th17/Th1 imbalance is increased in all patients that did not survive COVID-19.
[66]	Plasma (n = 384 plus C)	C = n.r ** P = 300	PEA	Relevant pathways associated with SARS-CoV-2 viremia (upregulation of virus entry factors, markers of lung tissue damage and coagulation) have been identified.
[67]	Plasma (n = 534)	**Development group** HC = 50 P = 100 **Validation group** HC = 78 P = 306	PEA	A 12-plasma protein signature and a model of 7 routine clinical tests as early risk predictors of COVID-19 severity and patient survival have been identified.
[68]	Plasma (n = 30)	HC = 10 P = 20 (10 ICU with severe/fatal pneumonia and 10 non-ICU with pneumonia)	UPLC-ESI-Q-Orbitrap-MS ELISA	A progressive increase in several complement cascade proteins and in inflammatory and platelet functions has been observed in COVID-19 patients.
[69]	Serum-derived EVs (n = 44 plus C)	HC = n.r. ** P = 44 (14 mild and 30 moderate/severe)	nLC-ESI-Q-Orbitrap-MS	Exploratory proteomic analysis of serum-derived EVs from patients with COVID-19 detected key proteins, associated with disease severity, related to immune response, coagulation activation and complement pathways.

* HC = healthy controls; C = controls; P = patients. ** not reported. Abbreviations: enzyme-linked immunosorbent assay (ELISA); capillary gel electrophoresis(CGE); laser-induced fluorescence (LIF); liquid chromatography (LC); nLC (nano LC); electrospray ionization (ESI); quadrupole (Q); mass spectrometry (MS); time of flight (TOF); chemiluminescence immunoassay (CLIA); ultra performance liquid chromatography (UPLC); matrix-assisted laser desorption ionization (MALDI); hydrophilic interaction liquid chromatography (HILIC); reverse transcription-quantitative polymerase chain reaction (RT-qPCR); nuclear magnetic resonance (NMR); Proximity Extension Assay (PEA); single-cell RNA sequencing (scRNA seq).

**Table 2:** List of articles dealing with multi-omics of serum/plasma in COVID-19 patients.

Reference	Source	Subjects Investigated *	Proteomic Technique	Main Findings
[72]	Serum (n = 118)	HC = 28 C = 25 (Patients with COVID-19-like symptoms negative to RT-PCR) P = 65 (37 non-severe and 28 severe)	LC-ESI-Q-Orbitrap-MS UPLC- ESI-Q-Orbitrap-MS	COVID-19 patients demonstrate characteristic changes in protein and metabolites associated with dysregulation of macrophage response, platelet degranulation, complement pathway signaling, and massive metabolic suppression.
[73]	Serum (n = 98)	C = 25 P = 73	UPLC-ESI-Triple Q-MS LC-ESI-Triple TOF-MS	Synthetic glucocorticoids in COVID-19 treatment modulate the neutrophil response.
[74]	Serum (n = 529)	HC = 125 P = 144 (108 non-severe and 36 severe)	LC-ESI-Q-Orbitrap-MS UPLC-ESI-Q-Orbitrap-MS LC-ESI-Triple TOF-MS	A high serum LDH level, due to hypoxia and tissue damage induced by inflammation, may be associated with higher COVID-19 severity.
[75]	Serum (n = 85)	HC = n.r. ** P = 85 (41 nonpulmonary fibrosis and 44 pulmonary fibrosis)	LC-ESI-Q-MS	PPAR signaling, TRP-inflammatory, immune system, and the urea cycle were pathways closely linked to the fibrosis formation and progression in patients with COVID-19.
[76]	Plasma (n ≥ 84)	HC = 30 P = 54 (30 non-severe and 24 severe)	UPLC-ESI-Q-MS UPLC-ESI-Q-Orbitrap-MS	COVID-19 survivors had altered extracellular matrix, immune response, hemostasis pathways, and lipid metabolism and changes in pulmonary fibrosis-related proteins.
[77]	Serum (n = 115)	HC = 27 C = 17 (patients with COVID-19-like symptoms negative to RT-PCR) P = 71 (48 non-severe and 23 severe)	UPLC-ESI-Q-Orbitrap-MS	The innate immune activation and inflammation triggered renal injuries in patients with COVID-19.
[78]	Serum (n = 99)	HC = 5 P = 46	Bioinformatics analysis	Three crucial pathways related to immunity and inflammation, including tryptophan, arginine, and glycerophospholipid metabolism, were considered to affect the effect of smoking on the adverse outcomes of COVID-19 patients.
[80]	Serum-EVs (n = 31)	HC = 9 P = 22	nLC-Orbitrap-MS scRNA-seq	MACROH2A1 is a potential biomarker candidate for refractory COVID-19 infection, and it may be involved in the pathogenesis of severe COVID-19 through its role in monocyte lineage and innate immunity.
[81]	Serum (n ≥ 103)	HC = n.r. ** P = 103 (40 mild, 34 severe, and 29 critic)	LC-ESI-Q-Orbitrap-MS GC-EI-Q-TOF-MS UPLC-ESI-Q-TOF-MS	Patients with the worst prognosis presented alterations in the TCA cycle (mitochondrial dysfunction), lipid metabolism, amino acid biosynthesis, and coagulation.
[82]	Serum (n = 30)	HC = 5 P = 25 (6 asymptomatic, 13 mild/moderate, and 6 severe)	RP-HILIC-LC-MS-based multi-omics analysis	Pregnancies with severe COVID-19 demonstrated greater inflammation and complement activation and dysregulation of serum lipids.
[83]	Plasma (n ≥ 82)	HC = 32 P = 50 (ICU)	PEA LC-MS/MS FIA-MS/MS LC-HRMS	Two proteins (CCL7 and CA14) and a lipid (HexCer 18:2; O2/20:0) showed improved sensitivity for predicting COVID-19 symptoms.

* HC = healthy controls; C = controls; P = patients. ** not reported. Abbreviations: gas chromatography (GC); electron ionization (EI); reverse phase (RP); flow injection analysis (FIA); high-resolution mass spectrometry (HRMS). For other abbreviations, see Table 1.
